# OpenSpineConsortium: An Open-Source Framework for Medical Student Engagement in Computational Spine Imaging Research

**DOI:** 10.7759/cureus.112661

**Published:** 2026-07-14

**Authors:** Ashley Schehr, Jerick Kim, Gregory Schwing

**Affiliations:** 1 Medicine, Wayne State University School of Medicine, Detroit, USA; 2 Surgery, Detroit Medical Center, Detroit, USA

**Keywords:** computational research, ct imaging, medical education, medical students, open-source, python programming, research training, spine imaging

## Abstract

Introduction: Research experience is heavily weighted in competitive specialties, yet preclinical schedules constrain student bandwidth. Prior work shows students acquire coding skills quickly and that imaging-based curricula engage them in scholarship, but programs are institution-bound and rarely couple anatomical learning with authentic computational contribution. This initiative combines open-source spine imaging datasets, Python coding, and a consortium model targeting a clinically high-impact anatomic region.

Methods: OpenSpineConsortium (OSC) is a distributed, open-source collaborative unifying public imaging collections into documented, openly licensed datasets, supporting anatomy-aware machine learning for the spine and pelvis. For medical students, this translated into analyzing spine CTs, annotating anatomical structures, and learning to automatically extract clinically useful parameters. Onboarding covers spine anatomy, segmentation, Python, and reproducible workflows. Students completed scoped sub-projects with senior-trainee mentorship. A post-participation survey assessed self-reported skill gains, scholarly output, and specialty interest.

Results: A total of 10 sub-projects span 16 contributors. Self-reported comfort (five-point scale) improved significantly across all six domains (Wilcoxon, all p<0.05), with the largest gains in CT spine interpretation (1.8-3.3, p<0.001), anatomic landmark/pathology identification (2.0-3.2, p<0.001), and Python coding (1.3-2.6, p<0.001). Conference submission, abstract writing, and literature reading also improved significantly. Eight of 16 reported increased interest in neurosurgery and 11 of 16 in research-oriented careers. All 16 would recommend OSC.

Conclusions: OSC produced significant gains in spine anatomy, computational proficiency, and scientific communication alongside 10 scholarly projects, supporting a scalable, low-cost model bridging anatomical education, computational training, and authentic contribution within medical school's time constraints.

## Introduction

Research experience is one of the most heavily weighted factors in residency selection across competitive specialties, and its importance has only grown since the transition of USMLE Step 1 to pass/fail scoring [1-3]. A national survey of program directors (PDs) found that surgical specialties were significantly more likely to rate research and scholarly work presentation as "extremely important" or "very important" compared with primary care specialties [1]. PDs across the most competitive specialties report that meaningful research participation will be even more important in offering interviews in the post-Step 1 pass/fail era, with 53.9% of the most competitive specialties endorsing this view compared with 33.1% of the least competitive [3].

Despite this demand, medical students face well-documented barriers to research engagement. Studies identified limited mentorship, heavy academic workload, and insufficient research training as the key barriers hindering medical students' engagement in research [4]. Time constraints and academic pressures are consistently among the most frequently cited obstacles [5,6]. These barriers disproportionately affect preclinical students, who must build foundational medical knowledge and assemble competitive residency applications simultaneously, leaving little bandwidth for traditional laboratory- or clinic-based research.

Several educational innovations have begun to address components of this gap. Programming and data science curricula have demonstrated that medical students can acquire computational skills rapidly, even without prior experience [7,8]. Similarly, low-cost virtual research workshops demonstrated significant improvements in research knowledge and self-efficacy, with research involvement increasing at six-month follow-up [9]. These findings collectively establish that medical students can develop technical and scholarly skills through structured programs that accommodate the demands of medical training.

Imaging-based curricula represent another promising avenue for engaging students in scholarship while reinforcing clinical knowledge. The Radiology Scholars Certificate Program, designed for preclinical students, combined shadowing, case-based modules, interactive workshops, and a capstone scholarly project, resulting in significant improvements in radiology knowledge and student confidence in ordering and interpreting imaging [10]. This educational gap is particularly relevant for spine imaging, where PDs have identified spine imaging interpretation as an area of perceived weakness requiring additional training [11]. The spine represents a clinically high-impact anatomic region where imaging literacy directly contributes to surgical rotation performance, yet structured educational opportunities for medical students in this domain remain scarce.

Despite these advances, existing programs that integrate computational training with imaging-based scholarship share important limitations. Most are institution-bound, requiring local infrastructure, faculty, and datasets that limit scalability and reproducibility [7,10]. Few programs couple anatomical learning with authentic computational contribution, where student work generates novel, reproducible analyses rather than serving purely didactic purposes. To our knowledge, no prior literature has identified targeted spine imaging as a vehicle for simultaneously developing radiographic literacy, computational proficiency, and scholarly output within a distributed, open-source framework.

OpenSpineConsortium (OSC) was designed to address these converging gaps. It is a distributed, open-source research collaborative that enables medical students to contribute to computational spine imaging research using publicly available CT datasets, Python programming, and version-controlled reproducible workflows. The consortium model is grounded in the communities of practice framework, in which novice learners acquire skills and professional identity through progressive, mentored participation in authentic tasks, and in self-directed learning theory, which has been associated with moderate knowledge gains over traditional teaching, particularly when learners are involved in choosing resources [12,13]. By combining anatomical education, computational training, and authentic scholarly contribution within a scalable model, OSC aims to bridge the structural mismatch between the research demands of competitive specialty applications and the time constraints of medical school. The primary objective of this study is program evaluation, characterizing OSC's feasibility and participant-reported outcomes rather than a definitive demonstration of long-term educational effectiveness, which would require controlled, longitudinal follow-up. This report describes the design, implementation, and initial evaluation of OSC, including self-reported skill gains, scholarly output, and specialty interest among the inaugural cohort.

## Materials and methods

Activity design and development

OSC was designed as an open-source research program enabling medical students to contribute to computational spine imaging research using publicly available datasets. The program draws on two primary datasets: VerseFusion (a full spine CT dataset) and CTSpinoPelvic1K (a dataset of annotated spine and pelvis CT scans). These datasets provide an important foundation for student-led sub-projects spanning clinically relevant preoperative metrics. Sub-project topics were proposed by students rather than assigned by mentors or derived from pre-existing laboratory research questions. Each student or small group independently identified a clinically meaningful spine or pelvic imaging metric of interest and proposed a corresponding computational pipeline; the program mentor reviewed each proposal for feasibility and scientific merit prior to approval but did not originate the topics. Students developed their pipelines using Python, with version-controlled workflows hosted on GitHub (San Francisco, California), and performed image visualization and segmentation using 3D Slicer (Brigham and Women's Hospital, Boston, Massachusetts) [14].

To support onboarding, a six-part AI medical imaging tutorial series was developed covering environment setup, medical image file formats, neural network inference, AI-assisted annotation using ITK-SNAP (Penn Image Computing and Science Laboratory, University of Pennsylvania, Philadelphia) [15], model training on institutional high-performance computing via SLURM, and scientific writing for AI imaging papers. All tutorials and coding workflows can be completed on a standard laptop using free resources; institutions without access to high-performance computing can substitute Google Colab's free GPU tier for the model training tutorial, making OSC implementable without specialized institutional infrastructure. An AI coding assistant workflow guiding students through development of computational analysis pipelines was also provided as a structured resource. This structure allowed students to pursue self-directed scholarly contributions while remaining within a mentored reproducible framework.

Claude (Anthropic, Sonnet 4.6; Anthropic, San Francisco, California) was used by students across sub-projects to assist with the development of Python code for image analysis pipelines, statistical analysis, and data visualization. All code, statistical analyses, and figures were generated using Python; Claude (Anthropic, San Francisco, California) assisted in writing this code. All AI-assisted outputs were validated against human measurements.

Curricular context and learner preparation

OSC is based at a single large, urban public medical school. Students were recruited via email outreach to the entire medical student body. No prior coding or imaging experience was required for participation. Upon joining, students were onboarded through a series of lab meetings conducted via Zoom (Zoom Video Communications, San Jose, California), which introduced foundational concepts in spine anatomy, CT image interpretation, medical image segmentation, and Python programming. One-on-one and small group mentorship sessions supplemented the group lab meetings and provided individualized support as students initiated and developed their sub-projects. This project was submitted to the authors' Institutional Review Board (IRB) for a Human Participant Research (HPR) determination. The IRB determined that this project does not meet the regulatory definition of human participant research and is not subject to IRB oversight; therefore, IRB review was not required.

Implementation

Following onboarding lab meetings, students worked independently and in small groups to develop and execute their computational pipelines. The program operated on a predominantly asynchronous model, allowing students to contribute on schedules compatible with preclinical coursework and clinical rotations. Synchronous events were the weekly lab meetings, which served as an opportunity for students to provide progress updates, assist with troubleshooting, and receive feedback on their projects. Additional one-on-one mentorship meetings were scheduled as needed based on individual student progress and project complexity. A total of 10 sub-projects were active at the time of survey administration, spanning a range of spine imaging metrics across the 16 participating students. Sub-projects spanned a range of clinically relevant spine imaging metrics, including automated Cobb angle measurement, sagittal lumbar lordosis quantification, lordosis distribution index calculation, vertebral Hounsfield unit analysis for opportunistic osteoporosis screening, lumbar intervertebral disc height measurement, pelvic incidence and sacral slope extraction, vertebral centroid trajectory analysis, vertebral wedging index computation, lumbar spinal stenosis screening via Torg ratio morphometry, and spondylolisthesis classification via Meyerding grading.

Assessment and evaluation

Program effectiveness was evaluated using a post-participation survey administered to all students following completion of their sub-project. The survey assessed self-reported comfort across six domains directly aligned with the program's educational objectives: CT spine image interpretation, anatomic landmark and pathology identification, Python coding, scientific abstract writing, conference submission, and critical literature reading. These domains were selected to reflect the core competencies targeted by OSC's curriculum (radiologic literacy, computational proficiency, and scholarly communication).

The survey consisted of six paired before-and-after items, each rated on a five-point Likert scale (one = not at all comfortable, five = very comfortable). The complete survey instrument is provided in Appendix A. Participants were asked to retrospectively rate their comfort in each domain both prior to and following participation. This retrospective pre-post design was selected to minimize response shift bias and capture perceived change attributable to the program. The survey instrument was developed by the study authors specifically for this evaluation and was not independently validated prior to use; face validity was established through mentor review of item wording against the program's stated educational domains. A one-tailed Wilcoxon signed-rank test was used because the study hypothesis specifically predicted improvement in comfort scores, not simply a change in either direction, consistent with the program's explicit educational aims. Additional items assessed specialty interest (neurosurgery and research-oriented careers), program satisfaction, and the likelihood of recommending OSC to peers. The survey was administered electronically at a single time point following completion of each student's sub-project contribution. All 16 participants completed the survey, yielding a 100% response rate. Statistical analysis was performed in Python using the Wilcoxon signed-rank test (one-tailed, testing for improvement) to assess pre-post differences within each domain, with significance defined as p<0.05.

## Results

Sixteen medical students participated in the OSC sub-projects and responded to the post-participation survey, representing a 100% response rate. The cohort was predominantly preclinical (12 M2, three M3, and one M4) with limited preparation: seven of 16 had no prior coding experience, and nine of 16 had only some incidental exposure (a class, tutorial, or dabbling). Eleven of 16 had no prior imaging experience, and four of 16 had viewing-only experience; only one of 16 had prior segmentation experience. Ten sub-projects were active across the 16 contributors at the time of survey administration. All 10 sub-projects were submitted as abstracts to a national neurosurgical conference, and several were additionally submitted to local institutional research symposia, representing concrete scholarly output.

**Figure 1:**
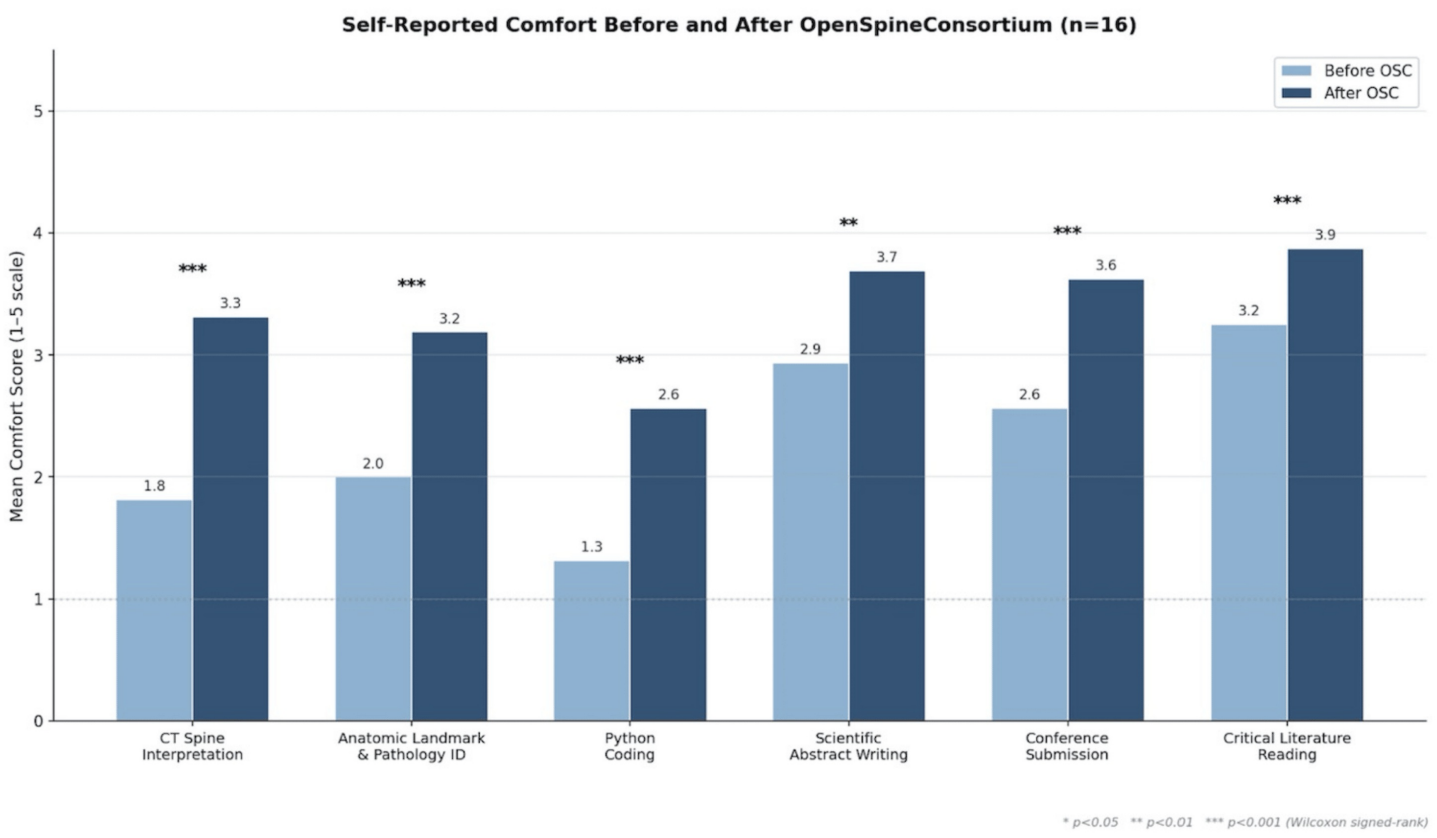
Self-reported comfort scores before and after participation in OSC (n = 16). Mean comfort ratings (one-to-five scale; one = not at all comfortable, five = very comfortable) are shown for six domains. Significance markers reflect Wilcoxon signed-rank tests comparing before and after scores. All six domains improved significantly (all p<0.05).

**Figure 2:**
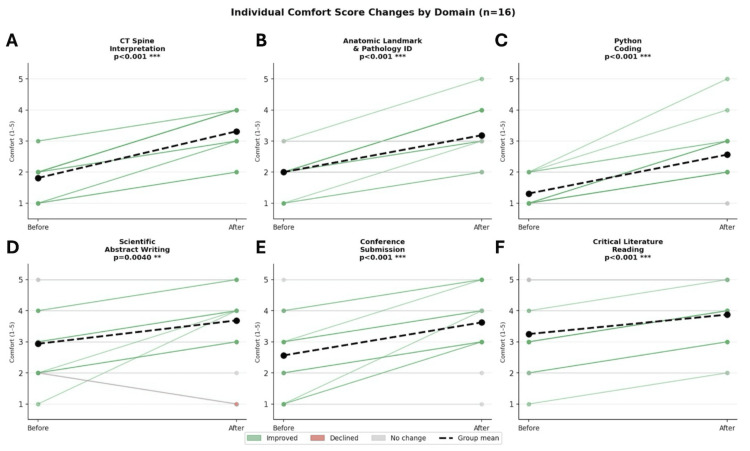
Individual-level comfort score trajectories before and after participation in OSC (n = 16). Shown by domain: CT spine interpretation (A), anatomic landmark and pathology identification (B), Python coding (C), scientific abstract writing (D), conference submission (E), and critical literature reading (F). Each line represents one student. Green lines indicate improvement, red lines indicate decline, and gray lines indicate no change. The dashed black line represents the group's mean trajectory. Significance markers reflect Wilcoxon signed-rank tests.

Self-reported comfort was assessed on a five-point Likert scale (one = not at all comfortable, five = very comfortable) across six domains before and after participation (Table 1). Significant improvement (p<0.05) was observed in all six domains (Figure 1). The largest gains were seen in CT spine interpretation (1.8-3.3, mean change = +1.5, p<0.001; improved in 16 of 16), Python coding (1.3-2.6, mean change = +1.3, p<0.001; improved in 13 of 16), and conference submission (2.6-3.6, mean change = +1.1, p<0.001; improved in 12 of 16). Anatomic landmark and pathology ID also improved significantly (2.0-3.2, mean change = +1.2, p<0.001; improved in 12 of 16), as did scientific abstract writing (2.9-3.7, mean change = +0.8, p=0.004; improved in 10 of 16) and critical literature reading (3.3-3.9, mean change = +0.6, p<0.001; improved in 10 of 16). Individual-level trajectories are shown in Figure 2. Effect sizes (matched-pairs rank-biserial correlation) were large across all domains, ranging from r = 0.85 (scientific abstract writing) to r = 1.00 (CT spine interpretation, anatomic landmark/pathology identification, Python coding, conference submission, and critical literature reading), the latter reflecting improvement in every participant who showed any change in that domain.

**Table 1:** Self-reported comfort scores before and after participation in OpenSpineConsortium (OSC) across six domains (n = 16). Mean scores reflect a five-point Likert scale (one = not at all comfortable, five = very comfortable). W-stat and p-values reflect Wilcoxon signed-rank tests (one-tailed, testing for improvement).

Domain	Before mean	After mean	Mean change	Improved (n)	W-stat	p-value
CT spine interpretation	1.81	3.31	+1.5	16	0.0	<0.001
Anatomic landmark and pathology ID	2	3.19	+1.19	12	0.0	<0.001
Python coding	1.31	2.56	+1.25	13	0.0	<0.001
Scientific abstract writing	2.94	3.69	+0.75	10	5.0	0.004
Conference submission	2.56	3.62	+1.06	12	0.0	<0.001
Critical literature reading	3.25	3.88	+0.62	10	0.0	<0.001

Open-ended survey responses highlighted themes consistent with the program's educational objectives. The most commonly cited valuable experiences included Python coding and AI-assisted workflow development ("Learning how to use resources such as AI to learn a completely new skill"; "Learning how to apply coding and imaging analysis to real clinical research projects"), CT spine image interpretation and segmentation ("Coding and reading spinal CTs"; "Reviewing images"), and research skill development ("Reading medical images and basic research writing/submission"). Several students also emphasized higher-order skills such as critical thinking and troubleshooting ("Being able to troubleshoot and think critically is a valuable skill that isn't used often during the preclinical years").

**Figure 3:**
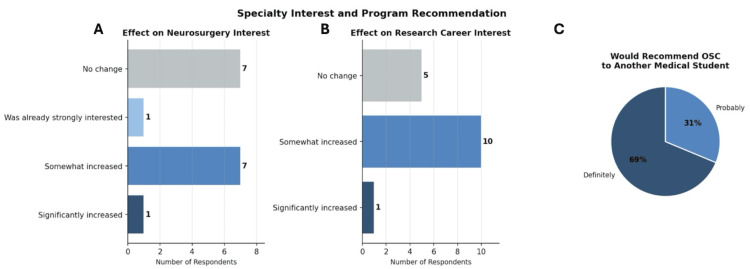
Specialty interest and program satisfaction among OSC participants (n = 16). Effect of participation on interest in neurosurgery (A) and research-oriented careers, including MD-PhD and surgeon scientist pathways (B). Respondents' likelihood of recommending OSC to another medical student (C). OSC: OpenSpineConsortium.

Eight of 16 participants reported increased interest in neurosurgery following participation, including seven who reported somewhat increased interest and one who reported significantly increased interest. Eleven of 16 reported increased interest in research-oriented careers (MD-PhD, physician-scientist), including 10 who reported somewhat increased interest and one significantly increased interest. All 16 respondents indicated they would recommend OSC to another medical student (Figure 3).

## Discussion

OSC produced significant self-reported gains across all six assessed domains, supporting the feasibility of a consortium model for engaging medical students in computational spine imaging research. The majority of the cohort entered with no prior coding experience and limited imaging exposure and demonstrated meaningful improvement in technically demanding skills, including CT spine interpretation and Python coding, after participation.

All 16 participants improved in CT spine interpretation, which is particularly notable given that 11 of 16 had no prior imaging experience at baseline. This indicates that structured engagement with segmentation tasks may serve as an effective mechanism for developing radiology interpretation skills in novice learners. Improvements in scholarly research skills, including scientific abstract writing, conference submission, and critical literature reading, suggest that OSC can serve as an effective outlet for developing research skills in preclinical medical students. Prior work has demonstrated that medical students can acquire research skills rapidly when involved in authentic projects [9], and OSC's model of scoped sub-projects with mentor oversight supports this. The submission of all 10 sub-projects to a national neurosurgical conference further demonstrates that self-reported improvements in abstract writing and conference submission translated into tangible scholarly outputs.

Qualitative feedback corroborated the quantitative findings, with students most frequently citing Python coding, AI-assisted workflow development, and CT spine interpretation as their most valuable experiences, alongside higher-order skills such as critical thinking and troubleshooting that are rarely practiced during preclinical training. The reported increases in interest in neurosurgery and research-oriented careers should be interpreted cautiously given the self-selected nature of the cohort and the absence of a comparison group. Students who self-selected to join a spine imaging consortium likely already have interest in surgical or research careers, which participation may expand rather than create.

This study has several limitations. It used a single-group pre-post design without a control group, and causal inference cannot be established. The sample size of 16 limits generalizability. In addition, the retrospective pre-post design is subject to recall bias, as participants rated their baseline comfort after completing the program. Self-reported comfort scores are subject to social desirability bias, and because no outcome measures were objectively assessed (e.g., via standardized coding tasks or blinded imaging interpretation tests), improvements in comfort may not correspond to measurable gains in actual competency. Because participation was entirely voluntary, the cohort likely reflects a self-selected subgroup of students who were already research-oriented or interested in surgical specialties and who may have had stronger baseline academic performance than the broader student body. This selection bias further limits the generalizability of observed skill gains and specialty-interest shifts to the general medical student population. Although all software tools used in OSC are free and open-source, successful implementation depended on mentor faculty with substantial expertise in Python, 3D Slicer, ITK-SNAP, SLURM-based high-performance computing, and medical imaging workflows. Institutions lacking access to similarly specialized mentorship may face practical barriers to replicating this model, independent of software cost. Future work should incorporate objective assessments of coding and imaging competency, a control group, longitudinal follow-up of career outcomes, and multi-institutional implementation.

## Conclusions

These findings support OSC as a scalable, low-cost model for integrating anatomical education, computational training, and authentic scholarly contribution into medical school, particularly during preclinical years. A program with no specialized infrastructure requirements produced significant gains across six skill domains and generated 10 nationally submitted conference abstracts among students with minimal prior technical experience. OSC addresses the gap that traditional research opportunities, often constrained by institutional access and scheduling demands, have been slow to fill. The open-source, distributed model offers a replicable framework for programs seeking to develop research-capable medical students without requiring substantial faculty time or institutional resources.

## Appendices

Appendix A: OSC program evaluation survey

Section 1: Skill Self-Assessment

For each of the following, rate your comfort before joining OSC and now on a five-point scale (one = not at all comfortable, five = very comfortable).

Item 1: Reading and interpreting CT spine imaging.

Item 2: Identifying spine anatomic landmarks and pathology.

Item 3: Writing Python code.

Item 4: Writing a scientific abstract.

Item 5: Submitting work to a scientific conference.

Item 6: Reading a primary research paper critically.

Section 2: Specialty and Career Impact

Item 7: What specialty were you considering before joining OSC?

Item 8: What specialty are you considering now?

Item 9: Has OSC affected your interest in neurosurgery? (Select one: Significantly increased; Somewhat increased; No change; Somewhat decreased; Significantly decreased; Was already strongly interested).

Item 10: Has OSC affected your interest in research-oriented careers (MD-PhD, surgeon-scientist, etc.)? (Select one: Significantly increased; Somewhat increased; No change; Somewhat decreased; Significantly decreased; Was already strongly interested).

Section 3: Open-Ended Questions

Item 11: What was the most valuable skill or experience you gained from OSC?

Item 12: What was the biggest barrier or challenge?

Item 13: Would you recommend OSC to another medical student? (Select one: Definitely; Probably; Maybe; Probably not; Definitely not).

Item 14: Any additional comments or suggestions?
